# Bidirectional relationships between depression, anxiety and urinary symptoms in women: A prospective cohort study

**DOI:** 10.1016/j.jad.2024.10.035

**Published:** 2024-10-10

**Authors:** Carol Joinson, Marcus J. Drake, Abigail Fraser, Kate Tilling, Jon Heron

**Affiliations:** a Bristol Medical School, Canynge Hall, 39 Whatley Road, Bristol BS8 2PS, United Kingdom of Great Britain and Northern Ireland; b Department of Surgery and Cancer, Imperial College London, South Kensington Campus, London SW7 2AZ, United Kingdom of Great Britain and Northern Ireland; c Bristol Medical School, Oakfield House, Oakfield Grove, Bristol BS8 2BN, United Kingdom of Great Britain and Northern Ireland

**Keywords:** Depression, Anxiety, Lower urinary tract symptoms (LUTS), Women, Prospective cohort study, ALSPAC

## Abstract

**Objectives:**

To examine (i) if depression and anxiety are prospectively associated with subsequent lower urinary tract symptoms (LUTS) and (ii) if LUTS are prospectively associated with subsequent depression.

**Participants and methods:**

The study is based on data from parous middle-aged women from the Avon Longitudinal Study of Parents and Children. LUTS were assessed using the Bristol Female LUTS Questionnaire and the International Consultation on Incontinence Questionnaire on Female LUTS. Depression was assessed using the Edinburgh Postnatal Depression Scale and anxiety was assessed using the Crown Crisp Experiential Index. We used multivariable logistic regression to examine (i) associations between depression and anxiety at baseline in 2002–04 and subsequent LUTS at follow-up in 2011–2012 (*n* = 5291) and (ii) associations between LUTS at baseline in 2002–04 and subsequent depression at follow-up in 2010–11 (*n* = 6147). Analyses were adjusted for age, socioeconomic factors, stressful life events, social support, smoking, weekly alcohol consumption, BMI, physical activity, obstetric/reproductive factors, and menopausal status.

**Results:**

We found evidence of prospective associations between depression and subsequent mixed urinary incontinence [odds ratio = 1.97, 95 % confidence interval = 1.16, 3.33], any urinary incontinence [1.68 (1.21, 2.31)], and urgency [1.90 (1.28, 2.83)]. Anxiety was only associated with subsequent nocturia [1.84 (1.04, 3.26)]. Only stress urinary incontinence was associated with subsequent depression [1.37 (1.03, 1.83)].

**Conclusions:**

We find evidence that mental health problems could be contributing factors, as well as consequences, of LUTS. Research is needed to determine if these observed associations are causal and to identify underlying mechanisms.

## 1 Introduction

Comorbidity of depression and anxiety with urinary incontinence (UI) is well-established in cross-sectional studies, but the direction of the association is unclear (Cheng et al., 2020). Around 10 % of adult women experience UI at least weekly, whilst 25 %–45 % report less frequent leakage (Altman et al., 2017). Stress UI is the most common subtype (estimated prevalence 10–39 %), followed by mixed UI (7.5–25 %), and urgency UI (1–7 %) (Altman et al., 2017). The wide variation in prevalence estimates is attributed to differing UI definitions and sample characteristics (e.g., age, ethnicity, parous versus nulliparous women).

It is often assumed that the elevated rates of mental health problems in women with UI are due to the perceived stigma of incontinence and its adverse consequences on the daily lives of affected people (Coyne et al., 2013). An alternative explanation, that mental health problems are a cause of incontinence, has also been suggested (Vrijens et al., 2015). Prospective studies that have focused on examining the direction of the relationship between depression/anxiety and UI have yielded inconsistent findings (Perry et al., 2006; Melville et al., 2009; Bogner et al., 2011; Legendre et al., 2015; Mishra et al., 2015; Felde et al., 2017). The inconsistencies might be due to studies not using validated questionnaires to assess UI; few studies differentiated between UI subtypes; some adjusted for only a small number of confounders, and some omitted important confounders such as parity, obstetric factors, and menopause status. Associations of depression and anxiety with other lower urinary tract symptoms (LUTS) including urgency and nocturia, have also been examined, but only in cross-sectional studies (Breyer et al., 2013; Vrijens et al., 2015).

The aim of the present study is to examine (i) if depression and anxiety are prospectively associated with LUTS and (ii) if LUTS are prospectively associated with depression. We hypothesise that (i) depression and anxiety increase the risk of subsequent LUTS and that (ii) LUTS increase the risk of subsequent depression. The study is based on data from a large UK population-based cohort of middle-aged women (mean age 41 years at the baseline for this study) and uses validated questionnaires to assess depression, anxiety and LUTS including UI subtypes (stress UI, urgency UI, mixed UI), urgency, and nocturia. Results are adjusted for a range of confounders, identified in empirical research and in consultation with clinical experts.

## 2 Participants and methods

### 2.1 Participants

The Avon Longitudinal Study of Parents and Children (ALSPAC) is a population-based cohort study which was established to understand how genetic and environmental characteristics influence health and development in parents and children. Mothers, partners and children have been followed up through regular questionnaires and clinics. The present study uses data from the ALSPAC mothers. ALSPAC recruited 14,541 pregnant women from the former Avon Health Authority in England, with an estimated delivery date between April 1991–December 1992 (Fraser et al., 2013; Boyd et al., 2013). Of the original 14,541 initial pregnancies, 338 were from woman who had already enrolled with a previous pregnancy, meaning 14,203 unique mothers were initially enrolled in the study. As a result of additional phases of recruitment, a further 630 women who did not enrol originally provided data since their ALSPAC study child was 7 years of age. This provides a total of 14,833 unique women enrolled in ALSPAC as of September 2021. Detailed information can be found at the cohort website (www.bristol.ac.uk/alspac), including a fully searchable data dictionary (www.bristol.ac.uk/alspac/researchers/our-data/).

Ethical approval for the study was obtained from the ALSPAC Ethics and Law Committee and the Local Research Ethics Committees. Specific details on the ethics committees and institutional review boards are available here: http://www.bristol.ac.uk/alspac/researchers/research-ethics/

### 2.2 Measures

#### 2.2.1 Depression

We used data from the Edinburgh Postnatal Depression Scale (EPDS) (Cox et al., 1987) to assess depression in the ALSPAC mothers in 2002–04 and 2010–11. The EPDS is a screening tool that was originally developed to measure symptoms of depression in the perinatal period but has been validated for assessing depression symptoms outside of this period. Participants select one of four options to reflect their feelings in the past week, such that a higher score reflects more severe depression symptoms. We defined depression cases as those with EPDS scores of 13 and higher because this threshold is often used to indicate probable depressive disorder (Evans et al., 2001).

#### 2.2.2 Anxiety

ALSPAC mothers also completed the anxiety subscale of the Crown Crisp Experiential Index (CCEI) (Crown and Crisp, 1979) in 2002–04 (at the same timepoint as EPDS completion). However, the anxiety assessment was not included by ALSPAC at 2010–11. The CCEI a validated self-rating inventory which asks participants to indicate, on a 4-point scale, how often they experience anxiety symptoms. There is no established threshold for the CCEI, but scores of 9 and more have previously been used to indicate high levels of anxiety.

#### 2.2.3 LUTS

Data on LUTS were collected from the ALSPAC mothers in 2002–04 and 2011–12 using the Bristol Female Lower Urinary Tract Symptoms (BFLUTS) and the International Consultation on Incontinence Questionnaire on Female LUTS (ICIQ-FLUTS) respectively (Brookes et al., 2004). The ICIQ-FLUTS was derived from the fully validated BFLUTS, which has been demonstrated to provide a valid and reliable assessment of LUTS [14]. The BFLUTS and ICIQ-FLUTS ask women to report their average frequency of LUTS over the past four weeks with options “*never*”, “*occasionally*”, “*sometimes*”, “*most of the time*”, and “*all of the time*”. We defined stress UI using the question “*Does urine leak when you are physically active, exert yourself, cough or sneeze?”*. Women who reported this symptom at least sometimes were defined as stress UI cases. We defined urgency UI using the questions “*Does urine leak before you can get to the toilet?”* and *“Do you have a sudden need to rush to the toilet to urinate?”*. Women who reported both symptoms at least sometimes were defined as urgency UI cases. Mixed UI cases were defined as women with both stress UI and urgency UI. To allow comparison with earlier studies, we additionally examined the presence of ‘any UI’ (i.e., women with any of stress UI, mixed UI, or urgency UI). We also examined a category that included women with any ‘urgency’ (i.e. irrespective of whether it was accompanied by UI). Furthermore, we examined nocturia and defined cases as women who reported waking to urinate at least twice a night because this frequency is considered clinically meaningful.

**Fig. 1:**
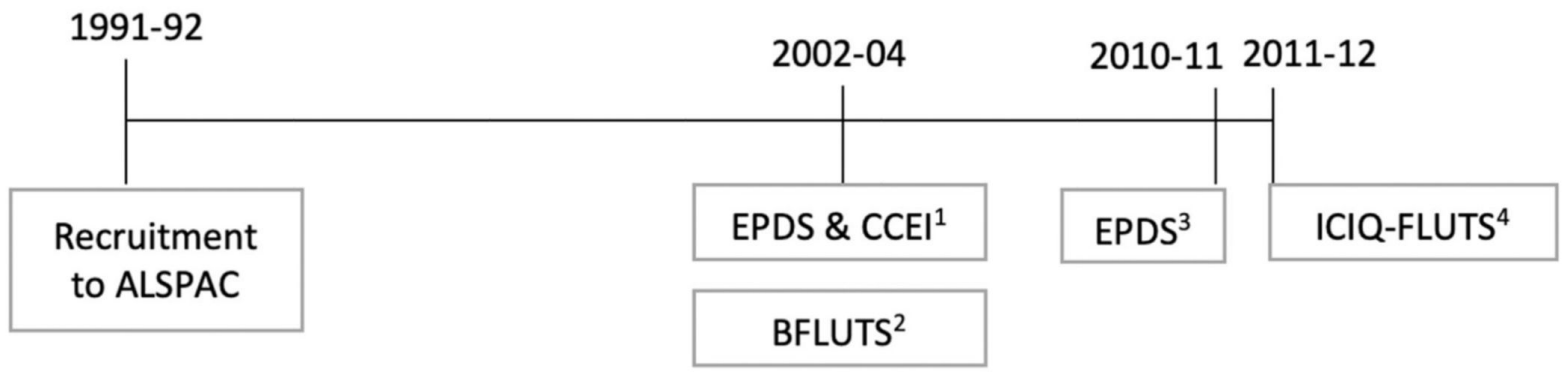
Timeline showing when LUTS, depression, and anxiety were assessed. 1. EPDS (Edinburgh Postnatal Depression Scale) & CCEI anxiety scale (Crown Crisp Experiential Index): depression and anxiety exposures in analysis (I) (prospective associations between depression/anxiety in 2002–04 and LUTS in 2011–12). 2. BFLUTS (Bristol Female Lower Urinary Tract symptoms): LUTS exposures in analysis (II) (prospective associations between LUTS in 2002–04 and EPDS in 2010–11). 3. EPDS: depression outcome in analysis II (prospective associations between LUTS in 2002–04 and EPDS in 2010–11). 4. ICIQ FLUTS (International Consultation on Incontinence Questionnaire on Female LUTS): LUTS outcomes in analysis I (prospective associations between depression/anxiety in 2002–04 and LUTS in 2011–12).

A timeline for the assessments of LUTS, depression, and anxiety is provided in Fig. 1.

#### 2.2.4 Confounders

We adjusted for participant age in 2002–04 (the baseline for this analysis); socioeconomic factors; stressful life events; social support; smoking; weekly alcohol consumption; BMI; physical activity; obstetric/reproductive factors, and menopausal status. See table S1 for details of all variables included the study.

### 2.3 Statistical analysis

**Fig. 2:**
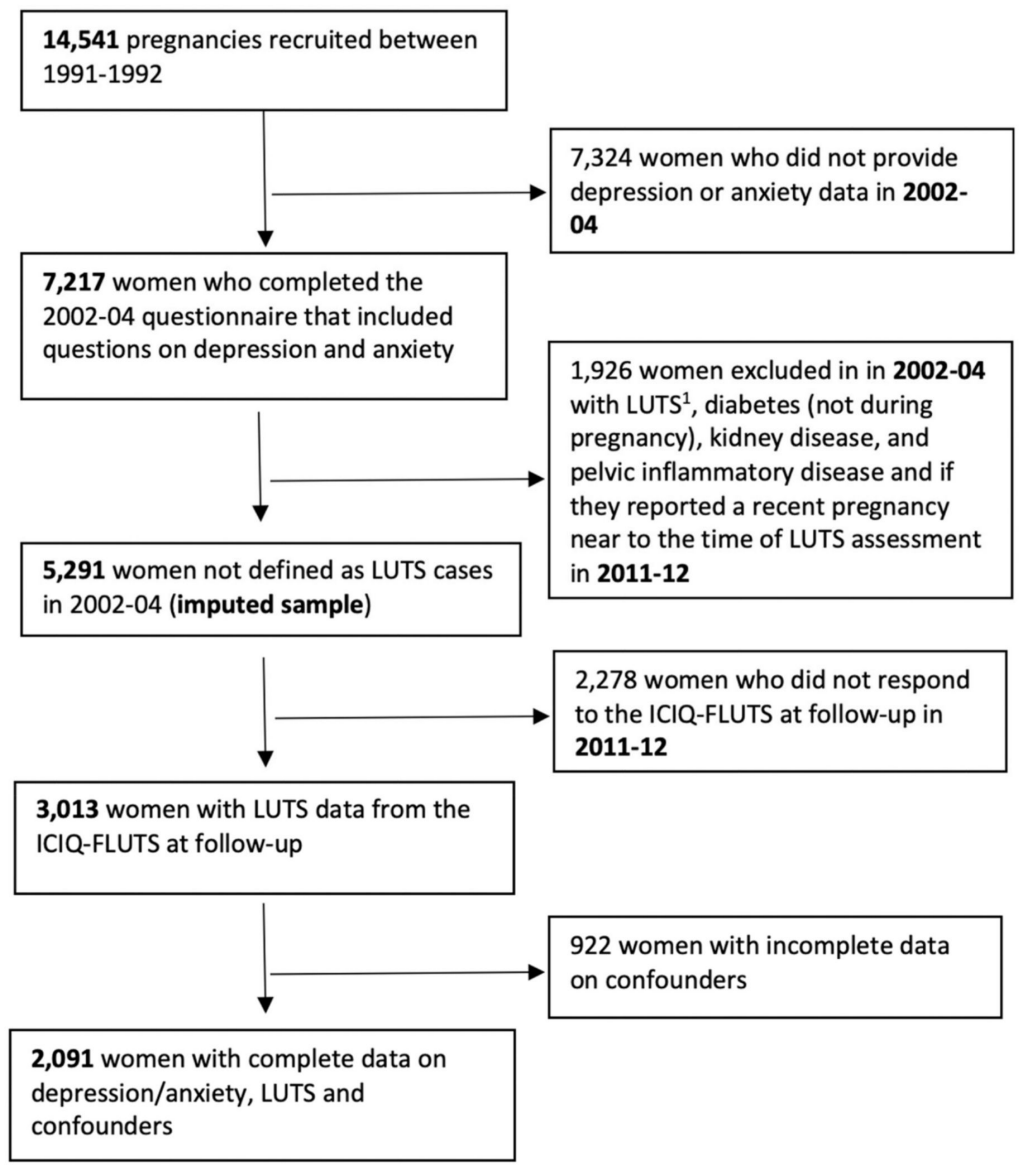
Flow chart showing the sample derivation for analysis (I): prospective association between depression and anxiety in 2002–04 and subsequent LUTS in 2011–12. 1. Urinary leakage before getting to the toilet, stress urinary incontinence, and urgency at baseline were defined as present if the following symptoms were reported to occur at least sometimes: “*Does urine leak before you can get to the toilet?*”, “*Does urine leak when you are physically active, exert yourself, cough or sneeze?*” “*Do you have a sudden need to rush to the toilet to urinate?*”. Nocturia (“*During the night, how many times do you have to get up to urinate, on average?*”) was defined as present at baseline if women reported passing urine ≥ 2 times nightly.

Analysis (I) – to examine if depression and anxiety (at baseline in 2002–04) are prospectively associated with subsequent LUTS (at follow-up in 2011–12): Fig. 2 shows the sample derivation for analysis (I). In accordance with the prospective cohort study design, we first excluded women who reported LUTS in 2002–04 and then classified them according to their exposure status (i.e. ‘depressed’ vs. ‘non-depressed’ and ‘anxious’ vs. ‘non-anxious’). We also excluded women who reported in 2002–04 possible organic causes of LUTS including diabetes (outside of pregnancy), kidney disease, and pelvic inflammatory disease, and women who reported being pregnant when LUTS were assessed in 2011–12. We used multivariable logistic regression analysis, adjusting for the confounders listed above, to examine if the odds of LUTS at follow-up were greater in women who reported depression or anxiety at baseline compared with those who did not have depression/anxiety. We also examined models that were mutually adjusted for depression and anxiety.

**Fig. 3:**
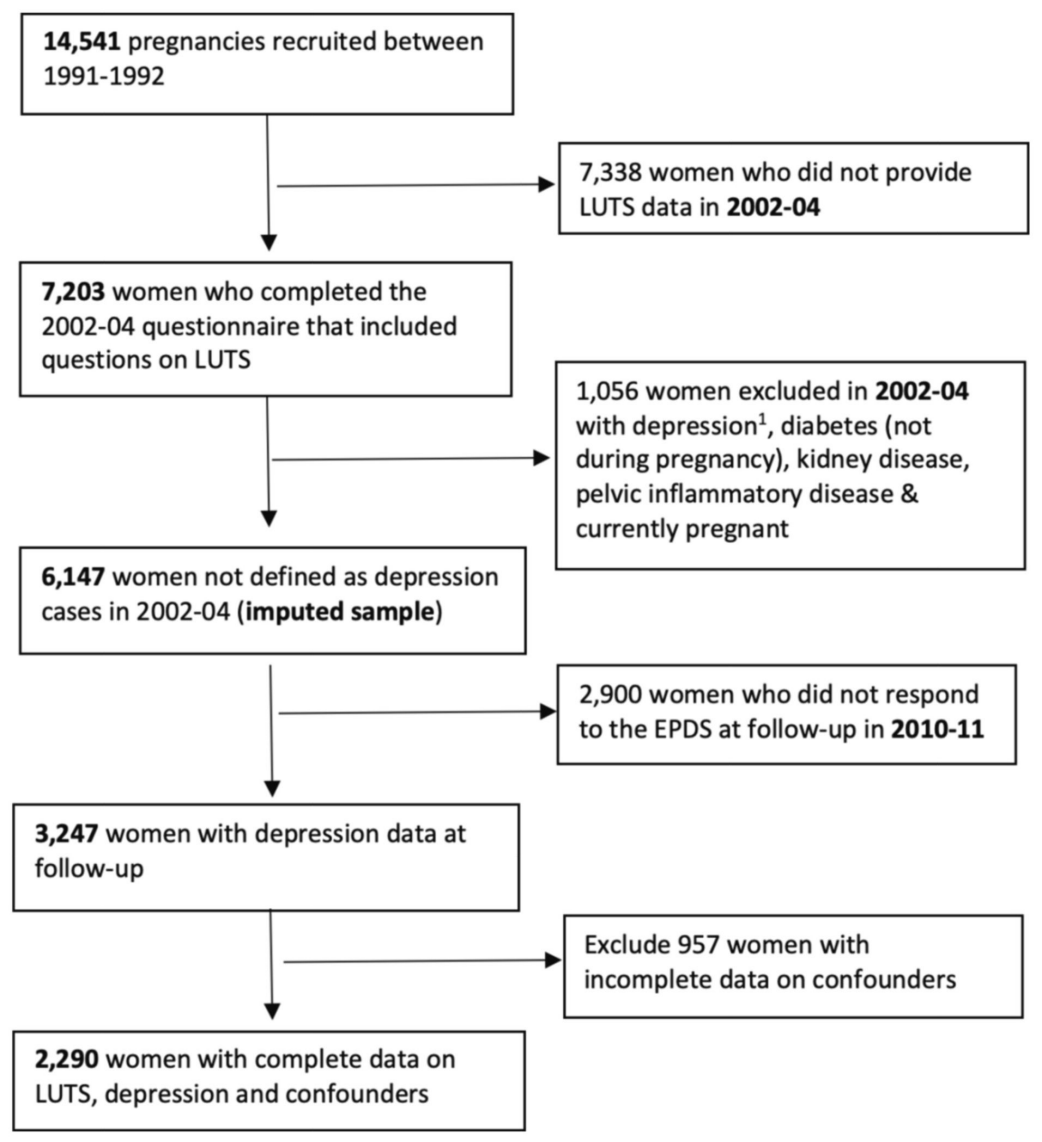
Flow chart showing the sample derivation for analysis (II): prospective relationships between LUTS in 2002–04 and subsequent depression in 2010–11. 1. Depression cases defined as EPDS score of ≥ 13.

Analysis (II) - to examine if LUTS (at baseline in 2002–04) are associated with subsequent depression (at follow-up in 2010–11): Fig. 3 shows the sample derivation for analysis (II). We first excluded women who had depression at baseline (2002–04) and then classified them according to their exposure status (i.e. ‘LUTS’ vs. ‘no LUTS’). We also excluded women who reported in 2002–04 having diabetes (outside of pregnancy), kidney disease, pelvic inflammatory disease, and being pregnant. We used multivariable logistic regression analysis, adjusting for the same confounders listed above, to examine if the odds of depression were greater in women who were exposed to LUTS compared with those who were unexposed to LUTS.

#### 2.3.1 Missing data

The amount of missing data for each variable in analysis (I) and (II) is provided in table S2. We used multivariable imputation by chained equations to address possible bias due to missing data (Sterne et al., 2009). We separately imputed missing data for analyses (I) and (II) using the mi impute chained command in Stata under the Missing at Random (MAR) assumption. For both analysis (I) and (II), we imputed 100 datasets with up to with 25 cycles of regressions switching. The results were combined using Rubin’s rules and we report the results from the imputed data as the main findings and compare these with the results from the complete case analysis in line with current recommendations (Sterne et al., 2009).

We conducted analysis (I) on an analysis sample of 5291 women who provided complete data on depression and anxiety and who were not defined as LUTS cases in 2002–04. We imputed missing data on LUTS in 2011–12 and missing confounders. Table S3 compares the descriptive statistics for depression and anxiety exposures in 2002–04, LUTS outcomes in 2011–12, and confounders in the analysis sample and the sample with complete data. In addition to variables in the main analysis, we included auxiliary variables that were likely to be related to the missing data mechanism in the imputation model. These included data on the presence of occasional LUTS and nocturia once a night in 2002–04, and data from earlier timepoints on daytime urinary frequency, problems holding urine when jumping/sneezing, constipation, UTIs, childhood UI, parental history of bedwetting, self-rated health, state-trait anxiety, indigestion, medication for depression/anxiety, oral contraceptives, earlier measures of the confounders, and additionally data on car access and home ownership status. We did not attempt to impute the exclusions described above therefore exclusions were made based on observed data. As a sensitivity analysis, we repeated analysis (I) on the sample of 2091 women who provided complete data on anxiety and depression in 2002–04, LUTS in 2011–12, and confounders.

We conducted analysis (II) on an analysis sample of 6147 women who provided complete data on LUTS and who were not defined as depression cases in 2002–04. We imputed missing data on depression in 2010–11 and missing confounders. Table S4 compares the descriptive statistics for LUTS exposures in 2002–04, the depression outcome in 2010–11, and confounders in the imputed and complete case datasets. In addition to variables in the main analysis, we included auxiliary variables that were likely to improve prediction of the variables being imputed. These included earlier measures of depression (EPDS), medication for anxiety/depression, and earlier measures of the confounders, in addition to data on car access and home ownership. We did not impute the exclusion indicators. As a sensitivity analysis, we repeated analysis (II) on the sample of 2290 women who provided complete data on LUTS in 2002–04, depression in 2010–11, and confounders.

## 3 Results

### 3.1 Analysis (I). Prospective associations between depression and anxiety in 2002–04 and subsequent LUTS in 2011–12

Table 1 shows the odds ratios and 95 % confidence intervals for the analysis of the associations between depression and subsequent LUTS.

Depression was associated with increased odds of mixed UI, any UI, and urgency after adjusting for confounders. The odds ratios for mixed UI and any UI were partly attenuated after further adjusting for anxiety, but there was still strong evidence of associations. The association between depression and urgency was strengthened after adjusting for anxiety. There was weak evidence that depression was associated with increased odds of urgency UI in the model adjusted for confounders and anxiety. We found no evidence for an association between depression and subsequent stress UI and nocturia.

Table 1 also shows the odds ratios and 95 % confidence intervals for the associations between anxiety and subsequent LUTS. Anxiety was associated with mixed UI and any UI in the models adjusted for confounders, but these associations were attenuated after further adjusting for depression. Anxiety was associated with subsequent nocturia after further adjusting for depression.

The complete-case analysis (Table S5) found weak evidence of an association between depression and any UI (after adjusting for confounders and anxiety), but the 95 % confidence intervals crossed the null in the model that was adjusted for anxiety. There was evidence that anxiety was associated with nocturia after adjusting for depression, but the 95 % confidence interval was wide.

### 3.2 Analysis (II). Prospective association between LUTS in 2002–04 and subsequent depression in 2010–11

Table 2 shows the odds ratios and 95 % confidence intervals for logistic regression analysis of the association between LUTS in 2002–04 and subsequent depression in 2010–11. Stress UI was associated with an increase in the odds of depression after adjusting for the confounders. Nocturia was also associated with an increase in the odds of depression in the unadjusted model, and there was weak evidence of an association in the adjusted model. There was little evidence that the other LUTS were associated with increased odds of depression.

In the complete-case analysis (Table S6), stress UI was associated with increased odds of depression in the adjusted model. There was also evidence that any UI was associated with depression in the unadjusted model, but the 95 % confidence interval crossed the null in the adjusted model.

As a post-hoc analysis, we also examined cross-sectional associations at baseline (in 2002–04) (Table S7). Both depression and anxiety showed strong cross-sectional associations with all LUTS, expect urgency UI.

## 4 Discussion

### 4.1 Main findings

We found evidence that depression is prospectively associated with mixed UI, any UI, and urgency (and weakly associated with urgency UI) after adjusting for confounders and further adjusting for anxiety. There was no evidence of an association between depression and subsequent stress UI or nocturia. The observed associations between anxiety and subsequent mixed UI and any UI were attenuated after further adjusting for depression, but anxiety was still associated with subsequent nocturia after adjusting for depression. When we examined prospective associations in the other direction (between LUTS and subsequent depression), stress UI was associated with subsequent depression, and there was weak evidence of an association with nocturia.

### 4.2 Strengths and limitations

The strengths of this study include the large community-based cohort; prospective design; validated questionnaires; different UI subtypes (in addition to urgency and nocturia), and adjustment for a wide range of confounders. Consistent with previous studies [1], we found that the proportion of women with urgency UI was smaller than the other subtypes, which resulted in less precision in the estimated associations. We did not examine daytime frequency since there is no minimum frequency that is accepted in the definition, and a high degree of overlap exists in what is perceived as normal versus abnormal (Drake, 2018).

The availability of both depression and anxiety data in 2002–04 enabled us to test for independent associations between depression and/or anxiety exposures and subsequent LUTS outcomes. However, care should be taken in the interpretation of these findings; different degrees of measurement error in the depression and anxiety exposures could potentially influence the findings of mutually adjusted models. Anxiety was not assessed in 2010–11 (or at any timepoints near this), hence we were unable to examine if LUTS are prospectively associated with anxiety.

There is evidence that depression, anxiety, and LUTS are more common in women from disadvantaged backgrounds (Lorant et al., 2003; Lee et al., 2020). Consequently, attrition bias due to selective dropout is a potential limitation because the sample with complete data included women who were more socioeconomically advantaged compared with the original ALSPAC cohort. Following current recommendations [17], we therefore reported the results from the imputed data as the main findings. There is evidence that multiple imputation eliminates bias regardless of the proportion of missing data (even with >50 % missing data) (Madley-Dowd et al., 2019).

In some cases we found only weak evidence of associations. It is important, however, to emphasise current recommendations that researchers should not base their conclusions purely on *p* value thresholds (e.g. *p* < 0.05) to determine statistical significance, but instead, consider the effect estimates alongside the strength of evidence indicated by the *p* values and confidence intervals (Amrhein et al., 2019).

The ALSPAC cohort is predominantly white and affluent (Fraser et al., 2013; Boyd et al., 2013) and hence we are unable to generalise our results to women from minority ethnic groups and less affluent populations. Further research in these underserved populations is vital to prevent widening inequalities in health research. Research in non-UK samples is also needed to examine if these findings generalise to women from other countries.

### 4.3 Potential mechanisms explaining the findings

There is some evidence that depression is associated with lower serotonin levels (Moncrieff et al., 2023), which have also been linked to increased detrusor overactivity (Okamoto et al., 2021). Drugs aimed at increasing serotonin levels have been found to improve overactive bladder (OAB) (Steers et al., 2007), whilst another study found that antidepressants are associated with an increased risk of UI (Felde et al., 2020). More recently, a randomised controlled trial showed that SSRIs are unlikely to cause or worsen stress UI (Christoffersen et al., 2022). More research is needed to understand the role of serotonin in the urinary system and to examine if antidepressants could be an effective treatment for UI.

Immune mechanisms, including oxidative stress and chronic inflammation, have been implicated as contributing factors for both depression and LUTS (Hughes Jr et al., 2023). HPA-axis dysregulation has also been suggested as a mechanism linking depression to OAB symptoms (Vrijens et al., 2015). A study found evidence that, compared with healthy controls, women with OAB have higher stress reactivity, suggesting that psychological stress exacerbates bladder urgency (Smith et al., 2016).

There is evidence to suggest that anxiety could be a contributing factor for nocturia, but the underlying mechanisms are unclear (Breyer et al., 2013). Nocturia is often observed in individuals with OAB and has also been attributed to reduced bladder capacity, and nocturnal polyuria (over-production of urine at night) (Smith et al., 2022). Nocturnal levels of urine production are mediated by arginine vasopressin (AVP), a hormone that results in decreased urine production, which is also thought to play a role in anxiety disorders (Caldwell et al., 2008). AVP production exhibits diurnal variation and normally increases during sleep as part of the normal circadian rhythm; there is evidence that anxiety disrupts this normal circadian rhythm (Coles et al., 2015).

Stress UI could increase the risk of depression due to its adverse effects on health-related quality of life, work productivity, and disruption to daily life (Chow et al., 2022).

## 5 Conclusion

Mental health problems are often ignored or inadequately assessed by clinicians who treat LUTS because their focus is on treating the bladder symptoms. Consideration is needed for a consensus on whether screening for depression and anxiety should be undertaken in women presenting with LUTS to ensure that mental health problems are assessed and treated. Clinicians treating women with depression and anxiety should be aware of the high level of comorbidity between affective disorders and urinary symptoms.

Research is needed to determine if the observed associations are causal. If subsequent research finds that depression and anxiety are causes of LUTS, this could justify changes in the current management of patients (e.g., antidepressants and/or anxiety interventions might be effective in alleviating LUTS). If stress UI causes depression, prevention efforts should be focussed on educating women about pelvic floor exercises, and to increase their awareness that stress UI may be preventable and not an inevitable symptom of aging. Non-causal relationships between depression/anxiety and LUTS are also important because it is well-established that poor mental health adversely affects treatment outcomes.

## Supplementary Material

Supplementary data to this article can be found online at https://doi.org/10.1016/j.jad.2024.10.035.

table Supplementary tables

## Figures and Tables

**Table 1:** Odds ratios (OR) and 95 % confidence intervals (CI) for the logistic regression analysis of the association between depression and anxiety in 2002–04 and subsequent lower urinary tract symptoms (LUTS) in 2011–12 (*n* = 5291).

	Stress UI	Urgency UI	Mixed UI	Any UI	Urgency	Nocturia
	OR (95 % CI)	OR (95 % CI)	OR (95 % CI)	OR (95 % CI)	OR (95 % CI)	OR (95 % CI)
Exposure = depression					
Unadjusted model	1.32 (0.91, 1.91)	1.97 (1.00, 3.88)	2.41 (1.65, 3.51)	1.90 (1.47, 2.44)	2.07 (1.55, 2.76)	1.24 (0.78, 1.98)
Adjusted for confounders^a^	1.34 (0.91, 1.97)	1.55 (0.75, 3.22)	2.05 (1.37, 3.06)	1.76 (1.35, 2.29)	1.73 (1.27, 2.35)	1.08 (0.66, 1.78)
Further adjusted for anxiety^b^	1.20 (0.77, 1.89)	1.97 (0.84, 4.65)	1.97 (1.16, 3.33)	1.68 (1.21, 2.31)	1.90 (1.28, 2.83)	0.75 (0.40, 1.40)
Exposure = anxiety					
Unadjusted model	1.32 (0.91, 1.93)	1.28 (0.61, 2.69)	1.92 (1.30, 2.84)	1.61 (1.24, 2.09)	1.51 (1.11, 2.04)	1.72 (1.12, 2.63)
Adjusted for confounders^a^	1.35 (0.91, 2.00)	0.95 (0.44, 2.07)	1.60 (1.06, 2.42)	1.47 (1.11, 1.94)	1.22 (0.89, 1.68)	1.58 (1.00, 2.50)
Further adjusted for depression^c^	1.21 (0.76, 1.92)	0.63 (0.25, 1.57)	1.07 (0.62, 1.85)	1.09 (0.77, 1.54)	0.84 (0.55, 1.26)	1.84 (1.04, 3.26)

The table shows the ORs and 95 % CIs for each type of LUTS in women with depression/anxiety compared to those without depression/anxiety (e.g., in the analysis with depression as the exposure and stress UI as the outcome, the OR and 95 % CI is for stress UI in women with depression compared to those without depression). Each logistic regression analysis was conducted on an imputed sample of *n* = 5291. All participants are included in all models.

a Adjusted for age, socioeconomic factors (occupational social class, educational attainment, material hardship), stressful life events, social support, ever smoker, weekly alcohol consumption, BMI, physical activity, parity, episiotomy/perineal tear, caesarean, menopausal status (see Table S2 in Supplementary Materials for the timing of assessment of each confounder).

b Adjusted for anxiety assessed at the same time as depression (in the questionnaire completed in 2002–04).

c Adjusted for depression assessed at the same time as anxiety (in the questionnaire completed in 2002–04).

**Table 2:** Odds ratios (OR) and 95 % confidence intervals (CI) for the logistic regression analysis of the association between lower urinary tract symptoms (LUTS) in 2002–04 and subsequent depression in 2010–11 (n = 6147).

		Depression
		OR (95 % CI)
Exposure = LUTS		
Stress UI	Unadjusted model	1.50 (1.14, 1.97)
	Adjusted model^a^	1.37 (1.03, 1.83)
Urgency UI	Unadjusted model	1.13 (0.54, 2.38)
	Adjusted model^a^	1.10 (0.51, 2.36)
Mixed UI	Unadjusted model	1.32 (0.84, 2.08)
	Adjusted model^a^	1.12 (0.69, 1.82)
Any UI	Unadjusted model	1.03 (0.99, 1.07)
	Adjusted model^a^	1.01 (0.97, 1.06)
Urgency	Unadjusted model	1.34 (0.99, 1.83)
	Adjusted model^a^	1.18 (0.85, 1.64)
Nocturia	Unadjusted model	1.84 (1.22, 2.78)
	Adjusted model^a^	1.53 (0.98, 2.39)

The table shows the ORs and 95 % CIs for depression in women with each type of LUTS compared to those without that type of LUTS (e.g., in the analysis with stress UI as the exposure and depression as the outcome, the OR and 95 % CI is for depression in women with stress UI compared to those without stress UI). Each logistic regression analysis was conducted on an imputed sample of *n* = 6147. All participants are included in all models.

a Adjusted for age, socioeconomic factors (occupational social class, educational attainment, material hardship), stressful life events, social support, ever smoker, weekly alcohol consumption, BMI, physical activity, parity, episiotomy/perineal tear, caesarean, menopausal status (see Table S2 in Supplementary Materials for the timing of assessment of each confounder).
