# Supplementary material for: Bidirectional relationships between depression, anxiety and urinary symptoms in women: A prospective cohort study
Source: J Affect Disord. Author manuscript; Available in PMC 2026 Apr 7. (PMC7618979; doi:10.1016/j.jad.2024.10.035)
Supplement: table Supplementary tables [file EMS207922-supplement-table_Supplementary_tables.docx]

**Table S1. Details of all variables used in the study: depression, anxiety, LUTS, confounders, and exclusions.**

| **Questionnaires / data collected & timing of assessment** | **ALSPAC variables** | **Question(s)/measure(s)** | **Final variable coding** |
| --- | --- | --- | --- |
| **Depression and anxiety assessed in 2002-04**    Edinburgh Postnatal Depression Scale (EPDS)                                                                                                                    Crown Crisp Experiential Index (CCEI) anxiety subscale | r4010              r4011            r4012            r4013            r4014            r4015            r4016            r4017            r4018            r4019              r4000 - r4007 | **EPDS items & response options**  I have been able to laugh and see the funny side of things  1 – As much as I always could  2 – Not quite so much now  3 – Definitely not so much now  4 - Not at all    I have looked forward with enjoyment to things  1 - As much as I ever did  2 – Rather less than I used to  3 – Definitely less than I used to  4 - Hardly at all    I have blamed myself unnecessarily when things went wrong  1 – Yes, most of the time  2 – Yes, some of the time  3 – Not very often  4 - Never    I have been anxious or worried for no good reason  1 – No, not at all  2 – Hardly ever  3 – Yes, sometimes  4 – Yes, often    I have felt scared or panicky for no good reason  1 – Yes, quite a lot  2 – Yes, sometimes  3 – No, not much  4 – No, not at all    Things have been getting on top of me  1 – Yes, most of the time I haven't been able to cope  2 – Yes, sometimes I haven't been coping as well as usual  3 – No, most of the time I have coped quite well  4 - No, I have been coping as well as ever    I have been so unhappy that I have had difficulty sleeping  1 – Yes, most of the time  2 – Yes, sometimes  3 – Not very often  4 - No, not at all    I have felt sad or miserable  1 – Yes, most of the time  2 – Yes, sometimes  3 – Not very often  4 - No, not at all    I have been so unhappy that I have been crying  1 – Yes, most of the time  2 – Yes, quite often  3 – Only occasionally  4 - Never    The thought of harming myself has occurred to me  1 – Yes, quite often  2 – Sometimes  3 – Hardly ever  4 – Never      **CCEI items**  Do you feel upset for no obvious reason?  Have you felt as though you might faint?  Do you feel uneasy and restless?  Do you sometimes feel panicky?  Do you worry a lot?  Do you feel strung-up inside?  Do you ever have the feeling you are going to pieces?  Do you have bad dreams which upset you when you wake up?    **Response options:**  1 – Very often  2 – Often  3 – Not very often  4 - Never | recode r4010 r4011 r4013 (1=0)(2=1)(3=2)(4=3)  recode r4012 r4014 r4015 r4016 r4017 r4018 r4019 (1=3)(2=2)(3=1)(4=0)  recode (-11=.) (-10=.) (-1=.)    EPDS sum score was computed by summing the scores for all items. Missing values were set to mode unless all values in were missing, in which case they were set to missing. A binary EPDS variable was derived with depression cases defined as those with EPDS scores>=13 and non-cases defined as those with EPDS scores <13.                                                                                                              recode r4000 r4005 r4006 (3=0) (4=0) (1=2) (2=2)  recode r4001 r4002 r4004 r4007 (4=0) (3=1) (1=2) (2=2)  recode r4003 (4=0) (1=2) (2=2) (3=2)  recode (-11=.) (-10=.) (-1=.)    CCEI sum score was computed by summing the scores for all items. Missing values were set to mode unless all values in were missing, in which case they were set to missing. A binary CCEI variable was derived with anxiety cases defined as those with CCI scores>=9 and non-cases defined as those with CCEI scores <9. |
| **Depression assessed in 2010-11**    EPDS | t3255 | EPDS sum score | recode (-10=.) (-1=.)  EPDS sum score was computed by summing the scores for all items. Missing values were set to mode unless all values in were missing, in which case they were set to missing. A binary EPDS variable was derived with depression cases defined as those with EPDS scores>=13 and non-cases defined as those with EPDS scores <13. |
| **LUTS assessed in 2002-04 and 2011-12**    Stress UI  Urinary leakage  Urgency  Nocturia        Stress UI  Urinary leakage  Urgency (with or without UI)  Nocturia                                  **Derived variables**  Stress UI  Urgency UI  Mixed UI  Any UI | r3004  r3003  r3002  r3001        U0030  U0050  U0040  U0020 | - Urine leaks when respondent is active /coughs/ sneezes - Frequency respondent leaks urine before she can get to toilet - Frequency respondent needs to rush to toilet to urinate - Number of times respondent gets up to pass urine at night          - Urine leaks when respondent is active /coughs/ sneezes - Frequency respondent leaks urine before she can get to toilet - Frequency respondent needs to rush to toilet to urinate - Number of times respondent gets up to pass urine at night     **Response options for Stress UI, Urinary leakage & Urgency:**  1 – never  2 – occasionally  3 – sometimes  4 – most times  5 – every time  -1 – missing    **Response options for nocturia:**  1 – none  2 – once  3 – twice  4 – three times  5 – four times or more  -1 – missing | - recode r3004 (3=1)(4=1)(5=1)(1=0)(2=0)(-1=.)(-10=.)(-11=.), gen(STRESS UI11) - recode r3003 (3=1)(4=1)(5=1)(1=0)(2=0)(-1=.)(-10=.)(-11=.), gen(UI11) - recode r3002 (3=1)(4=1)(5=1)(1=0)(2=0)(-1=.)(-10=.)(-11=.), gen(urgency 11) - recode r3001 (3=1)(4=1)(5=1)(1=0)(2=0)(-1=.)(-10=.)(-11=.), gen(nocturia11)          - recode U0030 (3=1)(4=1)(5=1)(1=0)(2=0)(-1=.)(-10=.), gen(STRESS UI) - recode U0050 (3=1)(4=1)(5=1)(1=0)(2=0)(-1=.)(-10=.), gen(UI) - recode U0040 (3=1)(4=1)(5=1)(1=0)(2=0)(-1=.)(-10=.), gen(urgency ) - recode U0020 (3=1)(4=1)(5=1)(1=0)(2=0)(-1=.)(-10=.), gen(nocturia)                                     gen STRESS UI=(STRESS UI==1)*(urgency ==0)  gen URGENCY UI=(UI==1)*(urgency ==1)*(STRESS UI==0)  gen MIXED UI=(STRESS UI==1)*(urgency ==1)  gen anyUI=(STRESS UI==1) \| replace anyUI=1 if UI==1 & urgency ==1 |
| **Confounders** |  |  |  |
| Age at delivery of index child (ALSPAC study child) | mz028b | Continuous variable: ages 15 - 44  -11 – triplet / quad  -4 –missing  -2 – miscarried | recode mz028b (-11=.) (-4=.) (-2=.), gen(ageatdelivery)  Use mz028b because more complete data. Add 12 to generate age at baseline for current study  gen age= ageatdelivery + 12 |
| Social class  1991 British Office of Population and Census Statistics job codes assessed in the antenatal period (1991-92) | c755, c765 | Derived variable from questions:   - Actual job, occupation, trade or profession - Please tick which of the following apply to you: foreman, manager, supervisor, leading hand, self-employed, none of these - Type of industry or service given (main things done in job)     **Response options**    1 - I  2 - II  3 – III (non-manual)  4 – III (manual)  5 - IV  6 - V  65 - Armed forces | Recode (1/3=0) (4/6=1) (else=.)    Select highest parental social class i.e., if one parent is non-manual then social class=0:    0: Non-manual: professional, managerial, or skilled professions  1: Manual: partly or unskilled occupations |
| Maternal education assessed in the antenatal period (1991-92)    Derived variable: mother’s highest educational qualification | c645a | 1: CSE/none  2: Vocational  3: O-level  4: A-level  5: Degree  -1: Missing | Recode (4/5=0) (3=1) (1/2=2)  0 = A-level or greater  1 = O-level  2 = CSE, vocational or less |
| Material hardship assessed in 1998-2000 | m5170 to m5174 | How difficult at the moment do you find it to afford these items:  a) food  b) clothing  c) heating*  d) rent or mortgage*  e) things you need for your children    1 Very difficult  2 Fairly difficult  3 Slightly difficult  4 Not difficult  5 – don’t pay for this (recode as 4) | foreach var of varlist m5170 m5171 m5172 m5173 m5174 {  recode `var' (1=1) (2=2) (3=3) (4=4) (5=4) (-11=.) (-10=.) (-1=.), gen(`var'r)  gen materialhardship_1 = 20 - m5170r - m5171r - m5172r - m5173r - m5174r    Mode imputation: Replace summed score =. with mode score if at least at least one of item answered    Score 0-15 (higher scores = more hardship) |
| Social support assessed in 2000-02    ALSPAC social support scale | p4020 - p4029 | Items assessing degree of perceived practical, emotional & financial support from others:    1 – Exactly feel  2 – Often feel  3 - Sometimes feel  4 - Never feel  7 – No partner | Summed score with reverse coding for some items:  Recode:  p4020: (1=0) (2=1) (3=2) (4=3)  p4021: (1=3)(2=2)(3=1)(4=0)(7=3)  p4024: (1=0)(2=1)(3=2)(4=3) (7=3)  p4026: (1=3)(2=2)(3=1)(4=0)(7=3)  p4022- p4023, p4025, p4027- p4029: (1=3)(2=2)(3=1)(4=0)  Sum all recoded items (higher scores = more social support) |
| Stressful life events assessed in 2002-04    ALSPAC life events inventory | r5000 - r5044 | Stressful events since child’s 9^th^ birthday (i.e. in 2000-02)  -11. Triplet / quadruplet  -10. Not completed  -1. No response    1. Yes, when study child was 9 or 10  2. Yes, since child's 11th birthday  4. No, did not happen in this period | Count life events occurring when ALSPAC study child was aged 9 or 10 (i.e. before mothers completed the depression/anxiety assessment when their child was aged 11- baseline for the current study).    Recode: (1=1) (2=0) (3=0) (4=0) (-11=.) (-10=.) (-1=.) |
| Smoking assessed in 1999-2001    (most recent measure of mothers’ smoking prior to 2002-04) | n5000 | Ever smoker  -11. Triplet / quadruplet  -10. Not completed  -1. No response  1. Yes  2. No | recode n5000 (-11=.)(-10=.)(-1=.) (1=1) (2=0)    Mother has ever been a smoker  1=yes  0= no |
| BMI: weight & height assessed in 2000-02 | p1290 p1291 | Self-reported weight and height  Continuous variables:  -11, -10, -1 = missing | Maternal BMI was derived from self-reported height and weight, as height (m)2 / weight (kg) |
| Physical activity assessed in 1999-2001 | n5110 n5120 | n5110 F9a: Amount of time mother spends jogging per week  n5111 F9b: Amount of time mother spends doing aerobics per week  n5112 F9c: Amount of time mother spends doing keep-fit exercises per week  n5113 F9d: Amount of time mother spends doing yoga per week  n5114 F9e: Amount of time mother spends playing squash per week  n5115 F9f: Amount of time mother spends playing tennis or badminton per week  n5116 F9g: Amount of time mother spends swimming per week  n5117 F9h: Amount of time mother spends walking briskly per week  n5118 F9i: Amount of time mother spends weight training per week  n5119 F9j: Amount of time mother spends cycling per week    1 >6 hours  2 2 - 6 hours  3 <2 hours  4 None  -1 missing | Women were asked to report the average hours in a typical week (>6 hrs, 2-6 hrs, < 2hrs, never) that they carried out different types of physical activity in 1999-2001 (8 years and 1 month after recruitment). Metabolic equivalents scores (METs) of the different activities were calculated using their estimated energy costs from the Compendium of Physical Activity (<https://sites.google.com/site/compendiumofphysicalactivities/>). Keep fit exercises and Other exercises were excluded because METs could not be assigned with confidence. MET-hours/week for each type of physical activity were derived by multiplying the hours/week spent doing the activity by the MET score for that activity. To derive the total physical activity score for each participant, MET hours per week were summed for the different types of physical activity. Since the response options of the time spent doing an activity (hrs/week) were categorial, hrs/week were estimated as follows:    6 for `≥ 6hrs', 4 for `2– 6hrs', 1 for `< 2hrs' and 0 for `never'. |
| Alcohol consumption assessed in 1999-2001 | n7250 - n7296 | During the last week how many of each type of alcoholic drink did you have on each  day?  Number of glasses of each drink on Mon, Tues, etc:  (i) Beer, lager or cider (no. of half pints)  (ii) Wine (no. of glasses)  (iii) Spirits (no. of single pub measures)  (iv) Other alcoholic drinks (please describe)  (no. of glasses or measures)  (vi) Low alcohol drink (no. of glasses or half pints) | Sum of each drink on each day to provide units consumed in the past week:  <https://journals.plos.org/plosone/article?id=10.1371/journal.pone.0167360>    Recode as missing: -11, -10, -9, -1    A small number of responses were coded:  97 - Sometimes, occasionally or <1  98 - Lots  71, 72, 81 - Cans, bottle  Recode all as missing since units cannot be calculated with confidence    Continuous score indicating how many of each type of drink on each day of the week. Compute sum of each drink on each day to provide units consumed in the past week |
| Episiotomy assessed 8 weeks after the birth of the index child (1991-92) | e251 | Painful stiches since birth  -1 – missing  1 – yes  2 - no | recode e251 (-1=.) (2=0)  1=yes  0= no |
| Caesarean: assessed 8 weeks after the birth of the index child (1991-92) | e041 | Had caesarean section  1- yes  2 - no  -1- missing | recode e041 (-1=.) (2=0),  1=yes  0= no |
| Parity assessed in the antenatal period (1991-92) | b032 | Number of previous pregnancies    -7, -2, -1 - missing  Continuous score (range 0-22) | recode b032 (-7=.) (-2=.) (-1=.) (0=0)(1=1)(else=2)    0 = no pregnancies previous to the current one  1 = 1 previous pregnancy  2+ = 2 or more previous pregnancies |
| Hysterectomy assessed in 2002-04 | r2083 | B6d: Reason why respondent has no periods:  -11, -10, -1 - missing  1 - They are pregnant  2 - Have had a hysterectomy  3- They are menopausal  4- Other reason  9 - Don't know | recode r2083 (-11=.) (-10=.) (-1=0) (1=0) (3=0) (4=0) (9=0) (2=1)    Recode ‘no response’ as still has periods    Hysterectomy  Yes=1  No=0 |
| Menopausal assessed in 2002-04 | r2083 | B6d: Reason why respondent has no periods:  -11, -10 – missing  -1 – no response  1 - They are pregnant  2 - Have had a hysterectomy  3- They are menopausal  4- Other reason  9 - Don't know | recode r2083 (-11=.) (-10=.) (-1=0) (1=0) (2=0) (3=1) (4=0) (9=0) (2=1)    Recode ‘no response’ as has not had hysterectomy    Menopausal  Yes=1  No=0 |
| **Other possible causes of LUTS** **assessed in 2002-04** |  |  |  |
| Pelvic inflammatory disease | r2016 | PID in the past year | Yes=1  No=0 |
| diabetes | r2030 -r2031 | Ever had diabetes (exclude cases with diabetes only during pregnancy) | Yes=1  No=0 |
| kidney disease | r2009 | Kidney disease in the past year | Yes=1  No=0 |
| Pregnancy | r2083 | Currently pregnant | Yes=1  No=0 |
| **Pregnancy assessed in 2010-11 (i.e. near to LUTS assessment in 2011-12)** | t4510 | Currently pregnant | Yes=1  No=0 |

**Table S2. Amount of missing information for each variable in analysis (I) prospective association between depression/anxiety and subsequent LUTS and analysis (II) prospective association between LUTS and subsequent depression and details of the imputation model**

|  | **Analysis (I)** | | **Analysis (II)** | |
| --- | --- | --- | --- | --- |
| **Variable** | **N missing (/5,291)** | **%** | **N missing (/6,147)** | **%** |
| **Mental health exposures** (in 2002-04) |  |  |  |  |
| Depression | 0 | 0 | - |  |
| Anxiety | 0 | 0 | - |  |
| **LUTS outcomes** (in 2011-12) |  |  |  |  |
| Stress UI | 2,270 | 43 | - |  |
| Urinary leakage | 2,271 | 43 | - |  |
| Urgency | 2,267 | 43 | - |  |
| Nocturia | 2,267 | 43 | - |  |
|  |  |  |  |  |
| **LUTS exposures** (in 2002-04) |  |  |  |  |
| Stress UI | - |  | 0 | 0 |
| Urgency UI | - |  | 0 | 0 |
| Mixed UI | - |  | 0 | 0 |
| Any UI | - |  | 0 | 0 |
| Any urgency (with or without UI) | - |  | 0 | 0 |
| Nocturia | - |  | 0 | 0 |
|  |  |  |  |  |
| **Mental health outcome** (in 2010-11) |  |  |  |  |
| Depression | - |  | 2,900 | 47.2 |

| **Confounders** |  |  |  |  |
| --- | --- | --- | --- | --- |
| Age | 0 | 0 | 0 | 0 |
| Manual social class | 339 | 6.4 | 418 | 7.9 |
| Maternal education | 118 | 2.2 | 145 | 2.7 |
| Material hardship score | 545 | 10.3 | 640 | 12.1 |
| Stressful life events score | 5 | 0.09 | 10 | 0.19 |
| Social support score | 600 | 11.3 | 733 | 13.9 |
| Ever smoker | 656 | 12.4 | 758 | 14.3 |
| Weekly alcohol consumption | 820 | 15.5 | 950 | 18 |
| BMI | 1,203 | 23.2 | 1,414 | 26.7 |
| Physical activity | 700 | 13.2 | 811 | 15.3 |
| Parity | 146 | 2.8 | 171 | 3.2 |
| Episiotomy | 234 | 4.4 | 258 | 4.9 |
| Caesarean | 229 | 4.3 | 252 | 4.8 |
| Menopausal | 0 | 0 | 0 | 0 |

**Table S3. Proportions and mean (SEs)) of depression and anxiety exposures assessed in 2002-04, LUTS outcomes assessed in 2011-12 and confounders in the analysis sample and sample with complete data**

|  | **Analysis sample (n=5,291)** | **Complete data**  **(n=2,091)** |
| --- | --- | --- |
|  | **% (SE) or mean (SE)** | **% (SE) or mean (SE)** |
| **Mental health exposures** |  |  |
| Depression | 9.0% (0.04) | 8.1% (0.06) |
| Anxiety | 9.0% (0.04) | 8.7% (0.06) |
| **LUTS outcomes** |  |  |
| Stress UI | 10.2% (0.06) | 10.4% (0.07) |
| Urgency UI | 1.9% (0.03) | 1.9% (0.03) |
| Mixed UI | 5.7% (0.04) | 4.8% (0.05) |
| Any UI | 17.8% (0.07) | 17.1% (0.08) |
| Urgency | 13.4% (0.07) | 11.7% (SE) |
| Nocturia | 6.3% (0.04) | 5.3% (0.05) |
| **Confounders** |  |  |
| Age (years) (in 2002-04) | 41.0 (0.06) | 41.7 (0.09) |
| Manual social class *(ref= non-manual)* | 14.3% (0.04) | 8.9% (0.06) |
| Maternal education *(ref= A level +)* |  |  |
| O levels | 35.9% (0.07) | 34.4% (0.01) |
| Vocational/CSE/none | 20.0% (0.06) | 12.4% (0.07) |
| Material hardship score | 1.5 (0.03) | 1.2 (0.05) |
| Stressful life events score | 2.6 (0.03) | 3.6 (0.08) |
| Social support score | 20.3 (0.07) | 20.5 (0.11) |
| Ever smoker *(ref=never smoker)* | 44.1% (0.07) | 37.3% (0.01) |
| Weekly alcohol consumption (units) | 5.9 (0.09) | 6.1 (0.13) |
| BMI (kg/m^2^) | 24.5 (0.06) | 24.2 (0.09) |
| Physical activity (MET hours/week) | 22.7 (0.28) | 23.2 (0.42) |
| Parity *(ref= 0)* |  |  |
| 1 | 36.4% (0.07) | 37.7% (0.01) |
| 2+ | 16.1% (0.05) | 14.2% (0.08) |
| Episiotomy *(ref= no episiotomy)* | 48.5% (0.07) | 51.3% (0.01) |
| Caesarean *(ref= no caesarean)* | 10.2% (0.04) | 9.8% (0.06) |
| Menopausal *(ref= not menopausal)* | 2.5% (0.02) | 2.3% (0.03) |

**Table S4. Proportions and mean (SEs)) of LUTS exposures assessed in 2002-04, depression outcome assessed in 2010-11 and confounders in the imputed and complete case datasets.**

|  | **Analysis sample (n=6,147)** | **Complete data**  **(n=2,290)** |
| --- | --- | --- |
|  | **% (SE) or mean (SE)** | **% (SE) or mean (SE)** |
| **LUTS exposures** |  |  |
| Stress UI | 9.6% (0.04) | 9.0% (0.06) |
| Urgency UI | 1.2% (0.01) | 1.2% (0.02) |
| Mixed UI | 3.7% (0.02) | 2.9% (0.03) |
| Any UI | 14.4% (0.04) | 13.7% (0.07) |
| Urgency | 10.1% (0.04) | 9.2% (0.06) |
| Nocturia | 4.4% (0.03) | 3.6% (0.04) |
| **Depression outcome** |  |  |
| Depression | 14.3% (0.06) | 13.4% (0.06) |
| **Confounders** |  |  |
| Age (years) (in 2002-04) | 41.1 (0.06) | 41.9 (0.09) |
| Manual social class *(ref= non-manual)* | 14.8% (0.05) | 8.8% (0.06) |
| Maternal education *(ref= A level +)* |  |  |
| O levels | 35.0% (0.06) | 33.8% (0.10) |
| Vocational/CSE/none | 21.9% (0.05) | 12.8% (0.07) |
| Material hardship score | 1.4 (0.03) | 1.1 (0.04) |
| Stressful life events score | 2.5 (0.03) | 2.6 (0.05) |
| Social support score | 20.4 (0.07) | 20.6 (0.10) |
| Ever smoker *(ref=never smoker)* | 44.5% (0.07) | 38.1% (0.10) |
| Weekly alcohol consumption (units) | 5.7 (0.08) | 6.0 (0.12) |
| BMI (kg/m^2^) | 24.8% (0.06) | 24.3 (0.09) |
| Physical activity (MET hours/week) | 22.5 (0.26) | 23.7 (0.41) |
| Parity *(ref= 0)* |  |  |
| 1 | 36.1% (0.06) | 37.1% (0.10) |
| 2+ | 17.1% (0.05) | 15.2% (0.07) |
| Episiotomy *(ref= no episiotomy)* | 48.2% (0.07) | 50.7% (0.01) |
| Caesarean *(ref= no caesarean)* | 10.0% (0.04) | 9.7% (0.06) |
| Menopausal *(ref= not menopausal)* | 2.7% (0.02) | 3.2% (0.04) |

**Table S5. Odds ratios (OR) and 95% confidence intervals (CI) for the association between depression and anxiety in 2002-04 and subsequent lower urinary tract symptoms ( LUTS) in 2011-12 (sample with complete data, n= 2,091)**

|  | **Stress UI** | **Urgency UI** | **Mixed UI** | **Any UI** | **Urgency** | **Nocturia** |
| --- | --- | --- | --- | --- | --- | --- |
| *Exposure= depression* |  |  |  |  |  |  |
| Unadjusted model | 1.38  (0.87, 2.20) | 2.92  (1.32, 6.43) | 1.75  (0.95, 3.20) | 1.79  (1.24, 2.57) | 1.71  (1.12, 2.60) | 1.29  (0.67, 2.45) |
| Adjusted for confounders^1^ | 1.37  (0.84, 2.22) | 1.97  (0.83, 4.65) | 1.51  (0.79, 2.88) | 1.61  (1.10, 2.35) | 1.37  (0.88, 2.13) | 1.05  (0.53, 2.07) |
| Further adjusted for anxiety^2^ | 1.33  (0.74, 2.39) | 2.41  (0.84, 6.89) | 1.30  (0.59, 2.88) | 1.55  (0.97, 2.47) | 1.47  (0.85, 2.53) | 0.68  (0.29, 1.55) |
| *Exposure= anxiety* |  |  |  |  |  |  |
| Unadjusted model | 1.28  (0.81, 2.03) | 1.89  (0.78, 4.57) | 1.62  (0.89, 2.97) | 1.52  (1.06, 2.20) | 1.37  (0.89, 2.11) | 1.87  (1.08, 3.26) |
| Adjusted for confounders^1^ | 1.23  (0.76, 1.99) | 1.18  (0.45, 3.09) | 1.52  (0.80, 2.88) | 1.36  (0.92, 1.99) | 1.10  (0.69, 1.73) | 1.67  (0.93, 3.02) |
| Further adjusted for depression^3^ | 1.05  (0.59, 1.89) | 0.69  (0.21, 2.57) | 1.31  (0.59, 2.88) | 1.06  (0.66, 1.70) | 0.88  (0.50, 1.54) | 2.05  (1.00, 4.20) |

The table shows the ORs and 95% CIs for each LUTS variable in women with depression/anxiety compared to those without depression/anxiety. Each logistic regression analysis was conducted on the complete case sample of n=2,091. All participants are included in all models.

1. Adjusted for age, socioeconomic factors (occupational social class, educational attainment, material hardship), stressful life events, social support, ever smoker, weekly alcohol consumption, BMI, physical activity, parity, episiotomy/perineal tear, caesarean, menopausal status (see appendix table 4 for the timing of assessment of each confounder).
2. Adjusted for anxiety assessed at the same time as depression (in the questionnaire completed in 2002-04).
3. Adjusted for depression assessed at the same time as anxiety (in the questionnaire completed in 2002-04).

**Table S6. Odds ratios (OR) and 95% confidence intervals (CI) for the association between lower urinary tract symptoms ( LUTS) in 2002-04 and subsequent depression in 2010-11 (sample with complete data n=2,290).**

| **LUTS** |  | **Depression** |
| --- | --- | --- |
|  |  | **OR (95% CI)** |
| Stress UI alone | Unadjusted model | 1.59 (1.10, 2.31) |
|  | Adjusted model^1^ | 1.50 (1.02, 2.21) |
|  |  |  |
| Urgency UI alone | Unadjusted model | 1.53 (0.58, 4.07) |
|  | Adjusted model^1^ | 1.46 (0.54, 3.97) |
|  |  |  |
| Mixed UI | Unadjusted model | 1.11 (0.54, 2.27) |
|  | Adjusted model^1^ | 0.93 (0.45, 1.95) |
|  |  |  |
| Any UI | Unadjusted model | 1.52 (1.10, 2.11) |
|  | Adjusted model^1^ | 1.40 (0.99, 1.96) |
|  |  |  |
| Any urgency (with or without UI) | Unadjusted model | 1.41 (0.93, 2.13) |
|  | Adjusted model^1^ | 1.27 (0.83, 1.94) |
|  |  |  |
| Nocturia | Unadjusted model | 1.58 (0.89, 2.80) |
|  | Adjusted model^1^ | 1.41 (0.78, 2.56) |

The table shows the ORs and 95% CIs for depression in women with each LUTS compared to those without the LUTS (e.g., in the analysis with stress UI as the exposure, the OR and 95% CI is for depression in women with stress UI compared to those without stress UI). Each logistic regression analysis was conducted on a complete case sample of n=2,290. All participants are included in all models.

1. Adjusted for age, socioeconomic factors (occupational social class, educational attainment, material hardship), stressful life events, social support, ever smoker, weekly alcohol consumption, BMI, physical activity, parity, episiotomy/perineal tear, caesarean, menopausal status (see appendix table 4 for the timing of assessment of each confounder).

**Table S7**. Odds ratios (OR) and 95% confidence intervals (CI) for the logistic regression analysis of the cross-sectional associations between depression, anxiety and LUTS in 2002-04 (n= 4186)

|  | **Stress UI** | **Urgency UI** | **Mixed**  **UI** | **Any UI** | **Urgency** | **Nocturia** |
| --- | --- | --- | --- | --- | --- | --- |
|  | **OR (95% CI)** | **OR (95% CI)** | **OR (95% CI)** | **OR (95% CI)** | **OR (95% CI)** | **OR (95% CI)** |
| *Depression* |  |  |  |  |  |  |
| Unadjusted model | 1.83 (1.40, 2.39) | 2.05 (1.08, 3.91) | 2.48 (1.70, 3.62) | 2.20 (1.76, 2.75) | 1.86 (1.43, 2.43) | 3.19 (2.33, 4.37) |
| Adjusted for confounders^1^ | 1.62 (1.23, 2.15) | 1.82 (0.91, 3.62) | 2.06 (1.38, 3.09) | 1.91 (1.51, 2.42) | 1.52 (1.15, 2.02) | 2.60 (1.85, 3.66) |
| *Anxiety* |  |  |  |  |  |  |
| Unadjusted model | 1.85 (1.43, 2.40) | 1.72 (0.89, 3.33) | 2.48 (1.72, 3.59) | 2.17 (1.75, 2.70) | 2.13 (1.65, 2.74) | 3.78 (2.80, 5.10) |
| Adjusted for confounders^1^ | 1.65 (1.25, 2.17) | 1.53 (0.76, 3.10) | 2.20 (1.48, 3.26) | 1.92 (1.52, 2.42) | 1.82 (1.39, 2.39) | 3.45 (2.48, 4.79) |

1. Adjusted for age, socioeconomic factors (occupational social class, educational attainment, material hardship), stressful life events, social support, ever smoker, weekly alcohol consumption, BMI, physical activity, parity, episiotomy/perineal tear, caesarean, menopausal status (see appendix table 4 for the timing of assessment of each confounder).
